# Noninvasive Assessment of Vascular Function: From Physiological Tests to Biomarkers

**DOI:** 10.1016/j.jacasi.2024.09.015

**Published:** 2024-11-19

**Authors:** Yukihito Higashi

**Affiliations:** Department of Regenerative Medicine, Research Institute for Radiation Biology and Medicine, Hiroshima University, Hiroshima, Japan; Division of Regeneration and Medicine, Medical Center for Translational and Clinical Research, Hiroshima University Hospital, Hiroshima, Japan

**Keywords:** arterial stiffness, biomarkers, endothelial function, vascular function

## Abstract

Vascular function is impaired by conditions such as hypertension, dyslipidemia, and diabetes as well as coronary risk factors including age, smoking, obesity, menopause and physical inactivity. Measurement of vascular function is useful not only for assessment of atherosclerosis itself but also in many other aspects such as understanding the pathophysiology, assessing treatment efficacy, and predicting prognosis of cardiovascular events. It is therefore important to accurately assess the extent of vascular function. A variety of vascular function assessments are currently used in clinical practice, including flow-mediated vasodilation, reactive hyperemia index, strain-gauge pulse plethysmographs, pulse wave velocity, augmentation index, intima media thickness, and chemical biomarkers. However, it is also true that there is no gold standard method for measuring vascular function in humans. To use vascular function effectively, it is necessary to understand the measurement-related pitfalls.

## Highlights

• Accurate assessment of vascular function is important for reducing the development of atherosclerosis, preventing cardiovascular events, and developing treatment strategies.
• In clinical practice, FMD, RHI, PWV augmented index, and various noninvasive assessment of vascular function, including chemical biomarkers, are carried out as vascular function assessment.
• To use vascular function effectively, it is necessary to understand the pitfalls associated with measurement.

The vascular system, consisting of the vascular system (arteries and veins) and the lymphatic system (lymph vessels), performs many life-supporting functions. The lumen of the vascular system is lined by a single layer of vascular endothelial cells forming the inner layer, with a tunica media composed of vascular smooth muscle cells (VSMCs) surrounding the vascular endothelial cells and an outer membrane located on the outer side. The vascular structure is strengthened by connective tissue and extracellular matrix. The 3-layered structure of the vasculature not only supports the structure of the vessels themselves, but also works to maintain their functions such as vasoconstriction and vasodilation. Classically, the venous system has been regarded simply as capacitance vessels, but the veins themselves may also have diverse functions.

Since the discovery of endothelium-derived relaxing factors secreted and produced by vascular endothelial cells in 1980 and the identification of nitric oxide (NO) as the main body of endothelium-derived relaxing factors in 1987, a vast body of basic knowledge relating to the vascular endothelium has accumulated.^1^, ^2^, ^3^, ^4^ In 1990, it was shown that vascular endothelial function was impaired in patients with hypertension.^5^ Risk factors such as hypertension, dyslipidemia, diabetes, aging, smoking, obesity, menopause, and physical inactivity have been shown to impair vascular function.^6^ Vascular function plays an important role in the onset, maintenance, and development of atherosclerosis.^7^, ^8^, ^9^ It is very important to accurately assess vascular function from a steady state to assess the degree of progression of atherosclerosis, treatment efficacy, and prognostic factors. Various attempts have been made to assess vascular function in a clinical setting. This review outlines the current status and future prospects for the evaluation of various vasoactive substances as surrogate markers from vascular function using physiological methods.

## Vascular Structure and Function

### Arteries

#### Vascular endothelial cells and vascular endothelial function

The vascular endothelium is located in the innermost layer of the vessel and consists of 1 cell layer. Although structurally, the vascular endothelium is a barrier separating the vascular lumen from the vessel wall, it produces and secretes a number of bioactive substances, including vasodilators, such as NO, prostaglandin I_2_, C-type natriuretic peptide, and endothelium-derived hyperpolarizing factor, and vasoconstrictors, such as endothelin, angiotensin II, prostaglandin H_2_, and thromboxane A_2_, which have exactly opposite effects. NO is produced and secreted by the endothelium through the stimulation of mechanosensors that sense shear stress caused by blood flow on vascular endothelial cells and by the binding of bioactive substances to their receptors.^10^, ^11^, ^12^ NO is produced and secreted from the essential amino acid L-arginine by activating endothelial nitric oxide synthase (eNOS).^13^ The secreted gaseous NO is transmitted by diffusion to neighboring VSMCs and activates soluble guanylate cyclase, which relaxes vascular smooth muscle via an increase in intracellular cyclic guanosine monophosphate levels.^14^ The normal vascular endothelium has vasodilation and contraction, VSMC proliferation and antiproliferation, coagulation and anticoagulation, inflammation and anti-inflammation, and oxidation and antioxidant effects, which work in balance to maintain vascular homeostasis.^9^ If vascular endothelial cells could be collected from the entire body, their total weight would be comparable to that of the liver, and if they could be spread over an entire surface, their total area would be equivalent to 6 tennis courts, or if they could be connected in a row, the length of the row would be about 100,000 km or 2.5 times around the earth.^15^

Vascular endothelial dysfunction is the first step in the development of atherosclerosis, maintaining and progressing atherosclerosis, and ultimately triggering cardiovascular events.^15^^,^^16^ So-called coronary risk factors, such as hypertension, diabetes, dyslipidemia, age, obesity, and smoking, as well as physical inactivity and menopause contribute to endothelial dysfunction.^6^ Aging is the most powerful determinant of endothelial function.^17^ Endothelial function has also been shown to be an independent determinant of the development of cardiovascular disease or cardiovascular events.^7^, ^8^, ^9^^,^^18^, ^19^, ^20^, ^21^, ^22^ Furthermore, endothelial function is a useful therapeutic target for atherosclerosis. Endothelial dysfunction can be restored by appropriate interventions such as pharmacotherapy, replacement therapy, and lifestyle modification.^23^, ^24^, ^25^, ^26^, ^27^ Improvement of endothelial function is expected to reduce the incidence of cardiovascular events and improve life expectancy.^28^, ^29^, ^30^, ^31^, ^32^, ^33^

The mechanisms that induce endothelial dysfunction have been elucidated, and various mechanisms have been postulated.^5^ Of particular interest is the reduction of biological activity of NO caused by reduced production of NO itself or inactivation of NO.^16^ Various mechanisms of reduced NO production, including abnormal receptors on endothelial cells, abnormalities in the signaling system after bioactive substances bind to the their receptors, reduced or abnormal shear stress, abnormalities in receptors that sense shear stress, abnormalities in endothelial NO synthase, and a lack of L-arginine as a substrate for NO, have been postulated.^6^ Although the lack of impairment of endothelium-independent vasodilation in the early stages of atherosclerosis rules out the involvement of reduced soluble guanylate cyclase activity or protein kinase G activity in VSMCs, abnormalities in VSMCs should also be considered under conditions of progressive atherosclerosis. NO inactivation is also important. It is known that NO trapping associated with increased production of reactive oxygen species (ROS), including activation of the renin-angiotensin system, inactivates NO.^28^ Furthermore, ROS not only inactivate NO but also combine with NO to convert it into peroxynitrite, which has very strong cytotoxic properties and impairs vascular wall cells, forming a vicious cycle, resulting in a reduction of the biological activity of NO in endothelial cells and VSMCs.^34^ Under oxidative stress conditions, NO not only induces endothelial dysfunction but also induces vascular smooth muscle proliferation, hypertrophy, and apoptosis, leading to vascular remodeling.^35^ Changes in vascular structure itself are assumed to be secondary to endothelial dysfunction. We showed that in patients with renovascular hypertension, there is a process of endothelial dysfunction caused by nicotinamide adenine dinucleotide phosphate oxidase activation associated with hyperactivity of the renin-angiotensin system, as well as excessive ROS production.^29^ As other mechanisms of endothelial dysfunction, endogenous NO synthesis inhibitors and an increase in various endothelium-derived vasoconstrictor substances may also be involved.

#### VSMCs and vascular smooth muscle function

VSMCs regulate vascular contraction, dilation and structural changes by binding to receptors for vasoactive substances secreted and produced in the blood or by endothelial cells, or by mechanical stimuli.^36^ Mature VSMCs have contractile and synthetic forms with a specific function for contraction. Although normal VSMCs synthase extracellular matrix and extracellular matrix-degrading enzymes to maintain vascular homeostasis, under the condition of atherosclerosis, they produce growth and migration factors to proliferate and migrate themselves, leading to the development of structural changes in the vasculature (pathological remodeling).^37^ VSMCs are transformed by external stimuli such as ROS and inflammation and become directly involved in the development, maintenance, and progression of atherosclerosis.^36^^,^^37^ Furthermore, transformed VSMCs form a vicious circle with atherosclerosis.

### Veins

The main physiological role of veins is to regulate circulating blood volume as capacitance vessels. The vascular system is an externally closed system and is maintained in circulatory equilibrium by cardiac output, which is the arterial system, and venous return. Approximately 75% of the circulating blood volume is retained in the venous system. The venous system is more extensible than the arterial system because of the lack of smooth muscle layers. A small change in internal pressure causes a large change in circulating blood volume, because blood flow increases by about 60% with each 1-mm Hg increase in venous pressure until extensibility reaches its limit. Venous return is mainly caused by the pumping action of the heart, with muscle pumping caused by contraction of the skeletal muscles themselves and respiratory pumping caused by increased intra-abdominal pressure during respiration also being involved in venous return. Although vasoactive substances and neurotransmitters may be involved in the regulation of intravenous pressure and venous return, their involvement is not considered to be significant.

### Lymphatic system

The lymphatic system (lymphatic vessels) is mainly responsible for the regulation of plasma protein levels and the immune system and also plays an important role as a transport system for cardiac vasoactive substances.^38^ Lymphatic vessels also have a 3-layered structure. The lymph fluid is mostly interstitial fluid leaking from capillaries. Interstitial fluid volume is regulated mainly by the balance between intracapillary pressure and plasma colloid osmotic pressure. Human lymphatic flow is estimated to be about 3 L/d and lymphatic intravascular pressure ranges from 0 to 4 mm Hg.^39^ Although details regarding the role of the lymphatic system in the cardiovascular system are unknown, it is thought that lymphatic vessels also regulate lymphatic flow and dilation/contraction by cardiac vasoactive substances.

## Vascular Function Measurements

**Figure 1:**
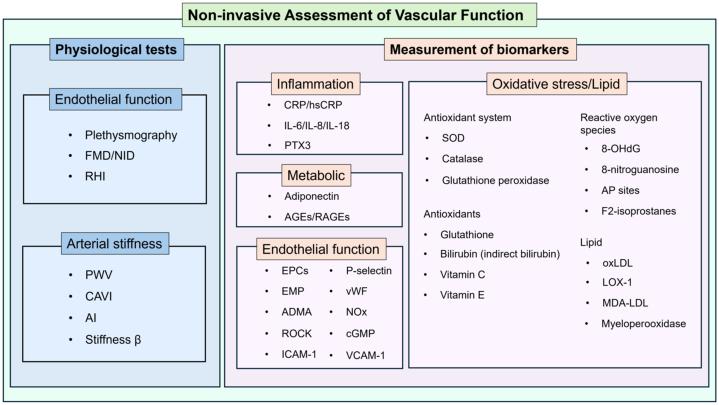
Noninvasive Assessment of Vascular Function Noninvasive assessment of vascular function: physiological tests and measurement of biomarkers are summarized in a clinical setting. 8-OHdG = 8-hydroxydeoxyguanosine; ADMA = asymmetric dimethylarginine; AGE = advanced glycation end product; AI = augmented index; AP = apurinic/apyrimidinic; CAVI = cardio ankle vascular index; cGMP = cyclic guanosine monophosphate; CRP = C-reactive protein; EMP = endothelial microparticles; EPC = endothelial progenitor cell; FMD = flow-mediated vasodilation; hsCRP = high-sensitivity C-reactive protein; ICAM = intracellular adhesion molecule; IL = interleukin; LOX = lectin-like oxidized low-density lipoprotein receptor; MDA-LDL = malondialdehyde-modified LDL; NID = nitroglycerine-induced vasodilation; NOx = nitrite/nitrate; oxLDL = oxidative low-density lipoprotein; PTX3 = pentraxin 3; PWV = pulse wave velocity; RAGE = advanced glycation end product receptor; RHI = reactive hyperemia index; ROCK = Rho-associated kinase; SOD = superoxide dismutase; VCAM = vascular cell adhesion molecule; vWF = von Willebrand factor.

Figure 1 and Central Illustration show the noninvasive assessment of vascular function from physiological vascular function measurements (eg, endothelial function and arterial stiffness) to measurements of biomarkers (eg, biomarkers of inflammation, metabolic substances, oxidative stress, lipids, and endothelial function) that are currently used.

**Central Illustration:**
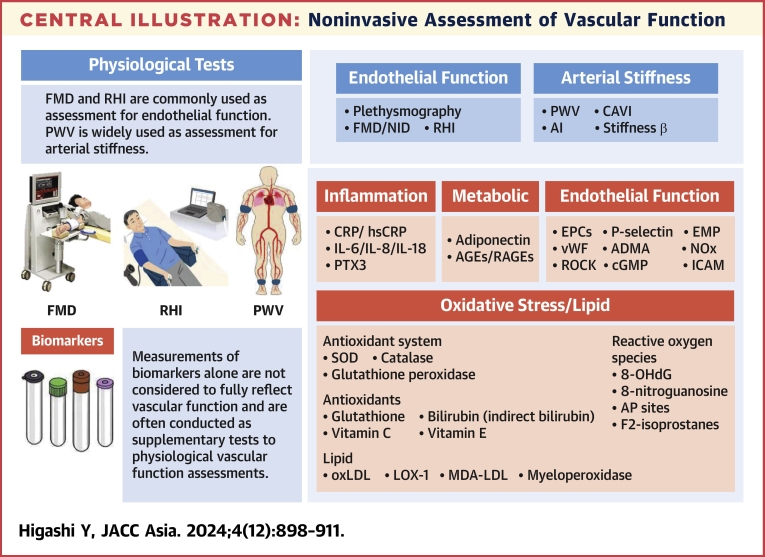
Noninvasive Assessment of Vascular Function Noninvasive assessment of vascular function: physiological tests and measurement of biomarkers are summarized. ADMA = asymmetric dimethylarginine; AGE = advanced glycation end product; AI = augmented index; AP = apurinic/apyrimidinic; CAVI = cardio ankle vascular index; cGMP = cyclic guanosine monophosphate; CRP = C-reactive protein; EMP = endothelial microparticles; EPC = endothelial progenitor cell; FMD = flow-mediated vasodilation; hsCRP = high-sensitivity C-reactive protein; ICAM = intracellular adhesion molecule; IL = interleukin; LOX = lectin-like oxidized low-density lipoprotein receptor; MDA-LDL = malondialdehyde-modified LDL; NID = nitroglycerine-induced vasodilation; NOx = nitrite/nitrate; oxLDL = oxidative low-density lipoprotein; PTX3 = pentraxin 3; PWV = pulse wave velocity; RAGE = advanced glycation end product receptor; RHI = reactive hyperemia index; ROCK = Rho-associated kinase; SOD = superoxide dismutase; vWF = von Willebrand factor.

### Endothelial function

Endothelial function measurements using physiological techniques have been reported.^30^ Flow-mediated vasodilation (FMD) and reactive hyperemia index (RHI) are methods for assessing blood flow and vessel diameter caused by postischemic reactive hyperemia. Plethysmography, in which a bioactive substance is administered and reactivity is assessed, and flow wires are also used to measure blood flow and vessel diameter.^40^, ^41^, ^42^ Measurements of vasoactive substances as surrogate markers are also expected in assessment of vascular function.^43^, ^44^, ^45^, ^46^, ^47^, ^48^, ^49^, ^50^, ^51^, ^52^, ^53^, ^54^, ^55^, ^56^, ^57^, ^58^, ^59^, ^60^, ^61^, ^62^, ^63^, ^64^, ^65^, ^66^, ^67^, ^68^, ^69^, ^70^, ^71^, ^72^, ^73^, ^74^, ^75^, ^76^

#### Strain-gauge plethysmography

Unfortunately, there is currently no standardized method for assessment of vascular endothelial function. Of the measurement methods that have been implemented, plethysmography is considered to be the most accurate method for assessing endothelial function.^5^^,^^6^^,^^23^, ^24^, ^25^, ^26^, ^27^^,^^29^^,^^40^, ^41^, ^42^ There are 3 methods for measurement using plethysmographs: pneumatic, photoelectric, and strain-gauge plethysmographs are used for assessment of endothelial function. In general, a thin strain-gauge filled with mercury is wrapped around the measurement site on the limb and the cuff is closed proximally to the measurement site to stop venous return, so that only arterial blood flows in and out at the measurement site. The difference in arterial blood flow causes a volume change in the limb tissue. Because these tissue volume changes are proportional to the circumferential diameter change at the limb measurement site, the blood flow can be measured by calculating the circumferential diameter change using a strain-gauge plethysmograph. By selective administration of vasoactive substances such as NO-producing stimulants or the NO synthesis inhibitor NG-monomethyl-L-arginine to the arteries of the limbs, blood flow changes can be measured to assess endothelial function.^23^^,^^24^ Measurement using a strain-gauge plethysmograph is thought to reflect endothelial function at the level of resistance vessels within skeletal muscle, based on the measurement site and principle of measurement. We have constructed a system based on blood flow measurement equipment (EC-5R, D.E. Hokanson) in combination with various auxiliary measuring devices.^24^^,^^29^ The method measurement using this system is highly specific because it evaluates various vasoactive substances by directly administering them into the arteries. However, it also has disadvantages such as a high burden on the examinee because of the insertion of a catheter into the limb artery and the lengthy examination time. Although a simpler method, which measures blood flow changes after reactive hyperemia, is also used, its specificity is lower than that of direct intra-arterial administration of vasoactive substances. Standardization of measurement methods and normal ranges of measured values have not been established for plethysmograph-based methods.

#### Flow-mediated vasodilation

Measurement of FMD is a method for assessing the change in vessel diameter after ischemia-responsive hyperemia in the limb by using ultrasound.^30^ FMD is calculated as: ([maximum vessel diameter after reactive hyperemia − baseline vessel diameter]/baseline vessel diameter) × 100, and is obtained as a percentage change. FMD can be measured with conventional ultrasound equipment, and dedicated FMD measurement equipment is also available.^14^ Based on the measurement site and principle, FMD is thought to reflect endothelial function at the conduit vessel level with a vessel diameter of around 2.5 to 5.5 mm. Measurement of FMD is simple and noninvasive with a relatively short test time and low burden on the examinee. Currently, measurement of FMD is the most widely used method for assessment of endothelial function. However, FMD measurement has a number of problems including reproducibility issues.

Increased shear stress after limb arterial depletion release triggers increased NO production, and piezo1 as a mechanosensor has recently been identified.^10^ In the hyperacute period immediately after limb arterial depletion release for several tens of seconds, an increase in intracellular calcium (Ca) concentration caused by the influx of Ca ions into the cell following the release of Ca-activated potassium channels may lead to increased NO production through eNOS activation.^31^ On a minute-by-minute basis after limb arterial depletion release, there is a Ca-independent Akt/PKB.^32^ Forearm blood flow increases to a maximum (300% to 600%) at around 5 to 10 seconds after limb arterial depletion release, and forearm vessel diameter increases to a maximum (5% to 15%) after delayed limb arterial depletion release of 45 to 60 seconds. Such phenomena strongly suggest that the transient increase in shear stress caused by limb arterial depletion release also releases NO and is involved in vasodilation. Indeed, in this series of responses, predosing with an NOS synthesis inhibitor does not alter the rate of increase in blood flow but markedly suppresses the increase in vessel diameter.^33^ With regard to NO-independent vasodilation, the involvement of factors such as adenosine and EDHFs has been postulated.

Although several FMD measurement guidelines have been proposed,^77^^,^^78^ no standardization of FMD measurement methods or normal values have been established; normal values for FMD range from 5% to 15%, depending on the report, and normal values for vasodilatation with nitroglycerine (nitroglycerine-induced vasodilation [NID]), an indicator of vascular smooth muscle function (endothelium-independent vasodilation), vary widely from 7% to 20%. Standardization of FMD measurements will enable assessment of therapeutic efficacy, elucidation of etiology and pathogenesis, and use as surrogate endpoints in large-scale clinical trials. Age, systolic blood pressure, obesity, male gender, and smoking are factors that decrease FMD, while an increase in heart rate is a factor that increases FMD.^79^ These confounding factors should be considered when measuring FMD. Baseline vessel diameter is also a potent confounding factor for FMD. Although FMD measurements are possible in the extremities, the characteristics of ultrasound make it difficult to measure FMD in vessels with diameters of <2.5 mm. FMD measurements are also unreliable when the measured arterial vasculature is larger than 5.5 mm. If possible, it is preferable to measure endothelium-independent vasodilation; the vasodilating capacity of sublingual administration of nitroglycerine is assessed as an endothelium-independent vasodilation. Although commonly used volumes of nitroglycerine are 300 to 400 μg per dose, the vasodilatory response at this volume is 15% to 20%, which is >5% to 10% of FMD. This dose of nitroglycerine relaxes vascular smooth muscle to a large extent. However, it is likely to be inappropriate as a control for FMD. In healthy subjects, it is advisable to use a dose of nitroglycerine that results in a vasodilatory response comparable to the vasodilatory response to FMD. To accurately assess vascular smooth muscle function, a volume response curve for nitroglycerine should be obtained and be analyzed. Furthermore, there is concern that nitroglycerine of normal volume may affect the vasodilatory response by decreasing body blood pressure and increasing heart rate. In our previous study, 75 μg of nitroglycerine administered sublingually produced vasodilation almost equivalent to that of FMD.^80^ In many studies, endothelial function has been assessed with the assumption that vascular smooth muscle function remains unchanged, although assessment becomes difficult when endothelium-independent vasodilation is altered by therapeutic intervention or other factors. We have assessed simultaneous changes in FMD and NID by calculating FMD/NID.^81^ It is also known that endothelial function changes with the menstrual cycle.^82^ FMD is significantly higher in premenopausal women in the follicular and luteal phases, when blood estradiol levels are high, than in the menstrual phase, when estradiol levels are low.^82^

In the measurement of FMD, the problem of reproducibility is one of the major challenges, as it depends largely on the skill of the operator, unless the measurement is fully automated.^33^ In manual measurement of FMD, more than 100 measurement experiences are required to be able to perform stable measurements, and more than 100 measurements per year are required to maintain the technique.^77^ Recently, the Japan Society for Vascular Failure has standardized the measurement method and set normal values based on measurements with a dedicated measuring device (UNEX EF, UNEX Co).^30^

#### Reactive hyperemia index

As a simple method, measuring finger volume pulse wave after reactive hyperemia has also been applied clinically.^83^, ^84^, ^85^, ^86^, ^87^, ^88^, ^89^, ^90^, ^91^, ^92^, ^93^ The method, which measures the fingertip volume pulse wave, is thought to reflect the vascular function of the skin tissue of the finger. The volume pulse wave of the apex artery is measured after reactive hyperemia using a dedicated probe. It has been shown that NO is involved in around 60% of RHI formation.^94^ RHI measurement is the simplest method for assessment of endothelial function because a fully automated measurement system is used and there are few differences between techniques. The Japan Society for Vascular Failure has standardized the method, and normal values have also been established on the basis of measurements made with a dedicated measuring device (Itamar Medical Ltd).^30^

#### Others

In the United States, the standard practice is to assess endothelial function by administration of vasoactive substances directly into the coronary arteries and measuring changes in vessel diameter by angiography and blood flow by using a flow wire.^95^ Measurement of blood flow with strain-gauge plethysmographs, FMD, and RHI is used as a surrogate for systemic endothelial function by measuring endothelial function in limb arteries. The method of measuring changes in vessel diameter and blood flow by direct intra-arterial administration of vasoactive substances, not only in coronary arteries, is highly specific. However, there is concern that the insertion of a catheter into the artery is a significant burden for the examinee. Endothelial function can also be assessed by measuring changes in blood flow in renal arteries induced by intravenous administration of L-arginine, a precursor of NO. Measurement of blood flow by the clearance method after intravenous administration of a vasoactive substance such as L-arginine has been used as a surrogate for endothelial function in renal arteries.^96^, ^97^, ^98^ However, there has been no study in which vasoreactivity was directly examined in renal arteries. To make the measurement quicker and simpler than measurements of conventional FMD, we have recently developed a new noninvasive device using the oscillometric method, plethysmographic FMD (Saraya Co), for assessing vascular response to reactive hyperemia in the brachial artery.^99^

### Arterial stiffness

Assessment of arterial stiffness is of great importance for elucidating the pathogenesis, etiology, and course of atherosclerosis and for formulating treatment strategies. Arterial stiffness is defined by the amount of vascular smooth muscle, elastic fibers (elastin), Ca deposition, and presence of atheroma. Clinically, arterial stiffness can be assessed by pulse wave velocity (PWV), cardio ankle vascular index (CAVI), and augmented index.

#### Pulse wave velocity

PWV is an index calculated from the distance between 2 points on an artery (L) and the time of pulse wave propagation between them (T). Because PWV is proportional to arterial wall stiffness, it reflects arterial stiffness. In Europe and the United States, PWV is measured as carotid-femoral pulse wave velocity (cfPWV), which is measured between 2 points, the carotid artery and the femoral artery.^100^, ^101^, ^102^, ^103^ From the measurement principle, the measurement site is from an unspecified point on the descending aorta to the femoral artery, and measurement of cfPWV is the gold standard for tests to assess aortic stiffness. In Japan and other Asian countries, the brachial-ankle pulse wave velocity (baPWV), which is measured between 2 points on the upper arm and ankle, is widely used.^104^, ^105^, ^106^ The method has few measurement sites in elastic arteries such as the aorta, and the majority of measurements are taken in muscular arterial sites such as the lower limb arteries; there are still questions as to whether the method accurately reflects arterial stiffness.

Although there has been no study clearly indicating a reference value for baPWV, a value of around 1,800 cm/s is considered appropriate as an index of arterial stiffness, based on indexes such as prediction of cardiovascular events.^106^ Also, for cfPWV, although there is no clear reference value, rough reference values have been set for each age, sex, and blood pressure value. It is considered to be around 1,000 cm/s as an indicator of arterial stiffness. Particular care should be taken when measuring baPWV. baPWV is low in cases of arteriosclerosis obliterans and aortic stenosis, and its accuracy is reduced in cases of arrhythmias such as atrial fibrillation. Standardization of measurement methods and normal values have also been established by the Japan Society for Vascular Failure, based on measurements with dedicated measuring instruments.^30^

#### Cardio ankle vascular index

CAVI was developed to measure arterial stiffness independent of blood pressure, whereas baPWV is strongly influenced by blood pressure.^107^^,^^108^ As with baPWV, the accuracy of CAVI measurements is compromised in cases of arteriosclerosis obliterans, aortic stenosis and regurgitation, and arrhythmias. A large-scale clinical trial using CAVI has been planned and is currently underway, and results are awaited. Standardization of measurement methods and normal values have also been established by the Japan Society for Vascular Failure, based on measurements with a dedicated measuring device (VaSera, Fukuda Denshi).^30^

#### Augmented index

Augmented index is the ratio of the driving pressure produced by the ejection of blood from the heart and the reflected pressure wave that is reflected back as the driving pressure wave travels through the cardiovascular system. Central artery pressure measurement methods required for augmented index calculation include catheter-based aortic pressure measurement (central pressure augmented index) and tonometric carotid artery pressure measurement (carotid augmented index). As a simple and noninvasive method, aortic pressure waveform is estimated from the pulse recorded in the radial artery and the augmented index is calculated (radial artery augmented index).^109^ A good correlation between radial artery augmented index and central artery augmented index has also been confirmed.

#### Stiffness β

Stiffness β is used for assessing arterial progression by carotid ultrasound echo and is calculated from carotid diameter change and blood pressure [β = ln(P/P)/(ΔD/D)].^110^ As shown in the formula, stiffness β is the arterial cross-sectional diameter and the percentage change in arterial cross-sectional diameter caused by blood pressure variation. Although it has the advantage that it is not affected by blood pressure, it is not versatile as it requires skilled techniques for measurement and long test times. In addition, stiffness β is thought to reflect local arterial stiffness from the measurement system and may not be suitable as an indicator of systemic arterial stiffness.

### Cardiovascular substances as biomarkers

In general, coronary risk factors and measurements of vascular function by various physiological techniques are used as surrogate markers of atherosclerosis. As surrogate markers of vascular function, measurement of biomarkers in blood or urine is the simplest and most noninvasive method, but, unfortunately, there are currently not enough biomarkers to withstand evaluation. Biomarkers of endothelial function include nitrite/nitrate, a metabolite of NO; NO second messenger cyclic guanosine monophosphate, and vascular cell adhesion molecule-1 and intercellular adhesion molecule (ICAM)-1, which reflect vascular endothelial damage; and plasminogen activator inhibitor-1 and von Willebrand factor (vWF), but these may not directly reflect NO production and are subject to various problems, including measurement accuracy. The measurement of these biomarkers should be considered as an adjunct to physiological methods for assessing vascular function. If biomarkers have high specificity as indicators of vascular function, they can be evaluated by measuring blood and urine levels, which would be of great benefit to the examinees and could be used in large-scale clinical trials and cohort studies.

#### Inflammation biomarkers

*C-reactive protein (CRP)/high sensitivity C-reactive protein (hsCRP):* CRP is mainly produced in the liver by inflammatory cytokines, but it is also produced locally in macrophages and VSMCs. CRP is the most widely used indicator of inflammation. In recent years, the usefulness of hsCRP as a biomarker for predicting the development of cardiovascular events has also been established.^43^, ^44^, ^45^ However, there are many negative views on whether CRP or hsCRP itself can be an independent prognostic factor for cardiovascular events.^46^^,^^47^ The standardization of assays has the advantage that they can be used, eg, in international collaborative studies.

*Interleukin (IL)-6*: IL-6 is a proinflammatory cytokine produced by many cells, including T cells, B cells, monocytes, macrophages, vascular endothelial cells, and adipocytes, and has a highly multifaceted action via binding to receptors. IL-6 increases the induction of ICAM-1 expression in vascular endothelial cells, stimulates macrophages to produce monocyte chemoattractant protein-1, acts as an inducer of CRP, enhances the induction of serum amyloid A, which is involved in inflammation-induced amyloid A amyloidosis, and enhances neutrophil function and migratory capacity.^48^ It is strongly involved in the maintenance and progression of inflammation by enhancing serum amyloid A induction and neutrophil function and migration. Indeed, high IL-6 levels have been reported in patients with angina pectoris, and IL-6 levels have also been associated with the development of cardiovascular events.^49^^,^^50^ In addition to IL-6, IL-8 mainly activates neutrophils and enhances their migratory potential, whereas IL-18 is produced mainly by activated macrophages and is involved in vascular injury by inducing the production of ROS and tumor necrosis factor-α via inducing the production of gamma interferons.^48^^,^^49^

*Pentraxin 3 (PTX3)*: PTX3 is an acute inflammatory protein that is expressed on vascular endothelial cells, VSMCs, leukocytes, and macrophages and is up-regulated under the condition of inflammatory stimuli.^51^ Circulating levels of PTX3 are known to be elevated in patients with unstable angina and are thought to play an important role in the formation of unstable plaques, particularly in coronary arteries.^52^ Some meta-analyses have revealed that PTX3 levels are associated with the development of cardiovascular events.^53^^,^^54^ PTX3 is expected to be a marker for ischemic heart disease such as unstable angina and myocardial infarction.

#### Oxidative stress biomarkers/lipid biomarkers

Unfortunately, there are currently no specific oxidative stress biomarkers. Therefore, there is also no biomarker specifically for the assessment of vascular function. However, many attempts have been made to establish oxidative stress biomarkers. These include direct measurement of ROS by spin trap methods, measurement of manganese-superoxide dismutase activity to assess SOD activity, which constitutes an antioxidant mechanism, measurement of catalase and glutathione peroxidase activity, and measurement of the antioxidants glutathione and bilirubin (indirect bilirubin).^55^ In addition, the following ROS and metabolites are measured: 8-hydroxydeoxyguanosine, 8-nitroguanosine, a nitrated nucleic acid of guanosine that is produced in the DNA damage process; apurinic/apyrimidinic sites, which are DNA base damage sites; and F2-isoprostanes, metabolites of the arachidonic acid cascade.^56^ NO itself is also classified as an ROS. Although associations of various measured oxidative stress markers with atherosclerosis have also been reported,^29^^,^^57^ whether they can be predictive markers for the development of cardiovascular events and long-term prognosis remains to be investigated.

#### Metabolic biomarkers

*Adiponectin*: Adiponectin is a bioactive substance secreted by adipocytes and is referred to as a bona fide adipocytokine. Adiponectin normally activates adenosine monophosphate-activated protein kinase and peroxisome proliferator-activated receptor α in various organs and enhances fatty acid burning and glucose uptake, and it has antiatherosclerotic and insulin-sensitizing effects.^58^ Adiponectin increases the biological activity of NO in endothelial cells and has anti-inflammatory and cardioprotective effects.^59^ Circulating adiponectin levels have been reported to be decreased in individuals with increased visceral fat mass, diabetes, and coronary artery disease.^60^^,^^61^

*Advanced glycation end products (AGEs)/AGE receptors:* AGEs bind to AGE receptors on vascular endothelial cell membranes, resulting in the activation of nicotinamide adenine dinucleotide phosphate oxidase and production of ROS.^62^ ROS have a very high binding affinity for NO and contribute to the inactivation of NO. Furthermore, the ROS produced are converted to peroxynitrite, which has a very strong cytotoxic effect when combined with NO.^63^ AGEs may also directly reduce the activity of eNOS. It has been reported that blood levels of AGEs are higher in patients with type 2 diabetes and are an independent predictor of the development of cardiovascular events.^64^ The presence of cardiovascular disease with increased AGEs, oxidative stress, and chronic inflammation forms a vicious cycle and results in endothelial dysfunction leading to the development of atherosclerosis.

#### Biomarkers of vascular endothelial function

It is no exaggeration to say that all of the aforementioned biomarkers are biomarkers of endothelial function. In a nonduplicative manner, we would like to describe the potential of biomarkers of endothelial function.

*Endothelial progenitor cells (EPCs)*: It is now clear that angiogenesis occurs through the differentiation and proliferation of EPCs recruited from bone marrow, and EPCs also play an important role in the re-endothelialization of damaged vessels. It has also been suggested that the number of EPCs in peripheral blood and their colony-forming capacity are a determinant of the development of cardiovascular events.^67^ The number of EPCs can be measured by flow cytometry. Although measurement of the number and function of EPCs is considered a promising biomarker for the assessment of endothelial function, the complexity of the procedure and the simplicity of the assay await improvement and development.

*Endothelial microparticles (EMPs):* Microparticles are released into peripheral blood from vascular endothelial cells, platelets, and leukemic cells by cell activation stimulated by various cytokines, physiological stimuli such as shear stress and hypoxia, and apoptosis.^68^ The microparticles released from vascular endothelial cells are collectively referred to as EMPs. Circulating levels of EMPs are known to be elevated in patients with cardiovascular events.^69^^,^^70^ EMPs themselves are assumed to reduce NO production, either directly or via disruption of the eNOS/NO pathway. EMPs are measured by flow cytometry, which is also a cumbersome and complicated procedure. A simple method for measurement of EMPs has not yet been established.

*Asymmetric dimethylarginine (ADMA)*: ADMA is an arginine derivative produced from arginine as a substrate. It is an L-arginine analogue and therefore has an antagonistic inhibitory effect on eNOS. Circulating levels of ADMA have also been reported to be elevated in groups with coronary artery disease or coronary risk factors.^71^ These results suggest that ADMA is involved in endothelial dysfunction through inhibition of the eNOS/NO pathway. ADMA can be measured relatively easily by both HPLC and ELISA methods.

*Rho-associated kinase (ROCK)*: ROCK is involved in increased vascular tonus, cell adhesion, cell proliferation and vascular remodeling in vessels by increasing Ca sensitivity of VSMCs. ROCK activation inhibits eNOS mRNA stabilization and suppresses Akt phosphorylation, thereby reducing eNOS activation.^72^ Clinically, ROCK activity has been assessed by measuring changes in vascular diameter and blood flow after intra-arterial administration of a ROCK inhibitor, but this method is highly invasive. The ratio between phosphorylation of myosin-binding subunits and total myosin-binding subunits in white blood cells as ROCK activity can also be measured by Western blot analysis.^73^ Although this method greatly reduces the burden on the examinee, it is still not a simple and accurate test.

*Soluble ICAM-1 (sICAM-1)/soluble P-selectin*: ICAM-1 is an adhesion factor expressed on vascular endothelial cells that ligands and binds to adhesion molecules expressed on white blood cells and mediates white blood cell adhesion to vascular endothelial cells. P-selectin is an adhesion factor expressed on the platelet membrane surface that leads to cellular interactions between platelets and white blood cells. Soluble adhesion molecules such as sICAM-1 and soluble P-selectin have been reported to be increased in patients with acute coronary syndrome.^74^

*vWF*: vWF is a protein produced by megakaryocytes and vascular endothelial cells and it plays a major role in thrombus formation by platelet aggregation and adhesion and in stabilizing coagulation factor VIII at the site of vascular injury.^75^ vWF levels have been shown to be increased in patients with arterial thrombosis and acute coronary syndrome, but the levels are also increased in patients with disseminated intravascular coagulopathy and thrombotic thrombocytopenic purpura and by exercise and stress.^76^

The methods currently used in clinical practice for assessing vascular function, including invasive testing, are shown in Table 1. It is needed to understand the methods currently used in clinical practice to assess vascular endothelial function, including their advantages, disadvantages, and measurement pitfalls, from the most widely used FMD, currently the most widely used laboratory-based but reliable plethysmography-based method, to the latest biomarkers.

**Table 1:** Assessments of Vascular Function Used in Clinical Practice

Measurement Site	Methods	Stimuli	Advantages	Disadvantages
Forearm artery Lower limb artery	By plethysmograph Blood flow measurement	Vasoactive agents Reactive hyperemia	High specificity due to direct intra-arterial administration of vasoactive agents Low burden on the examines (short test time, noninvasive) Simple	The burden on the subject is high (long examination time). The procedure is complicated (invasive). Somewhat lacking in specificity
Forearm artery	Ultrasound-based vascular diameter measurement (FMD) Oscillometric vascular diameter measurement (pFMD)	Reactive hyperemia Reactive hyperemia	Low burden on the examinee (short test time, noninvasive) Simple Low burden on the examinee (short test time, noninvasive) Simple	Somewhat lacking in specificity Somewhat lacking in specificity
Fingertip artery	Measurement by tonometry (RHI)	Reactive hyperemia	Low burden on the examinee (short test time, noninvasive) Simple	Somewhat lacking in specificity
Coronary artery	Blood flow measurement by a flow wire Angiographic measurement of vessel diameter	Vasoactive agents	High specificity due to direct intra-arterial administration of vasoactive agents	The burden on the subject is high (long examination time) The procedure is complicated (invasive)
Renal artery	Blood flow measurement by the clearance method	Vasoactive agents	Relatively low burden on subjects	Slightly less specific due to intravenous administration The procedure is complicated
Blood/urine	Measurement of concentrations of vascular endothelium-related substances (biomarkers)		Simple	Ancillary role to the above measurement methods due to low specificity

FMD = flow-mediated vasodilation; pFMD = plethysmographic flow-mediated vasodilation; RHI = reactive hyperemia index.

### Relationships between physiological vascular function and biomarkers

Biomarkers known as markers of endothelial function (eg, EPCs, EMPs, ADMA, ROCK, sICAM-1, soluble P-selectin, vWF, and nitrite/nitrate) have been identified by showing a correlation between FMD and RHI.^29^^,^^46^^,^^57^^,^^65^^,^^66^^,^^71^^,^^73^^,^^111^, ^112^, ^113^, ^114^, ^115^, ^116^ As a representative, the number of EPCs in peripheral blood and their colony-forming capacity are inversely correlated with FMD in healthy men.^66^ Circulating levels of ADMA are inversely correlated with the degree of endothelial dysfunction assessed by FMD and RHI in patients with dislipidemia.^71^ Leukocyte ROCK activity inversely correlated with FMD in smokers, healthy men, and patients with atherosclerosis,^111^, ^112^, ^113^ and ROCK activity assessed by fasudil infusion is inversely correlated with acetylcholine-induced vasodilation in the brachial artery.^73^ Changes in sICAM-1 are correlated with changes in FMD after resveratrol supplementation in patients with cardiovascular disease.^114^ Other biomarkers including inflammation biomarkers, oxidative stress biomarkers, lipid biomarkers, and metabolic biomarkers, but not all biomarkers, are associated with physiological vascular function.^46^^,^^73^^,^^115^ There are many negative views on whether CRP or hsCRP itself can be an independent prognostic factor for vascular function.^46^ PTX3, which is also an inflammatory marker of vascular endothelial localization, has been reported to have a stronger inverse correlation with FMD than hsCRP.^116^ Associations of various measured oxidative stress markers with vascular function have also been reported.^29^^,^^57^ Circulating levels of AGEs are inversely correlated with FMD in patients with type 2 diabetes.^65^

### Significance of the assessment of physiological vascular function and biomarkers as independent markers to predict future cardiovascular outcomes

Endothelial function has been shown to be a predictor of cardiovascular events in both clinical trials and meta-analysis.^7^^,^^9^^,^^18^, ^19^, ^20^, ^21^, ^22^^,^^29^, ^30^, ^31^, ^32^, ^33^ Because endothelial function is strongly associated with known traditional cardiovascular risk factors and encompasses these risk factors in a phenotypic manner, it may be self-evident that endothelial function is a predictor of cardiovascular events.^17^^,^^79^ Despite publication bias, almost all reports showed a significant association with all-cause mortality, especially cardiovascular disease-related death, in individuals with higher PWV including baPWV, suggesting that PWV may be a useful prognostic factor in cardiovascular events.^117^^,^^118^ Blacher et al^119^ reported that a 1-m/s increase in PWV is associated with a 39% increase in cardiovascular mortality. However, studies using physiological vascular function including FMD, RHI, and PWV have been conducted in high-risk populations such as patients with hypertension, patients with coronary artery disease, and patients with heart failure, and their predictive ability in the general population is not clear, so it is difficult to generalize them. Some but not all biomarkers have also been reported to predict cardiovascular events.^29^^,^^46^^,^^47^^,^^49^^,^^50^^,^^53^^,^^54^^,^^57^^,^^60^^,^^61^^,^^64^^,^^67^^,^^71^^,^^120^ However, it is also true that there is currently no specific biomarker that predicts future cardiovascular events. Both physiological vascular function and biomarkers have been reported to be predictors for future cardiovascular events, but no certain view has yet been reached. It is expected that a combination of physiological vascular function with biomarkers would be more useful for predicting future cardiovascular events. Future findings regarding whether or not physiological vascular function and biomarkers can predict future cardiovascular events in large clinical trials are awaited.

## Conclusions

There is no doubt that measurement of vascular function is of great clinical relevance. Many methods of measurement of vascular function have been tried, but it is important to be familiar with the advantages and disadvantages of each method, especially the measurement pitfalls, to accurately measure vascular function. Guidelines for the assessment and management of vascular function, including standardization of methods for measuring vascular function using physiological methods, have been issued.^30^^,^^77^^,^^78^ However, the content of the guidelines needs to be brushed up, including revision of the guidelines and consideration of biomarkers. Further accumulation of knowledge on the assessment of vascular function is awaited.

## Funding Support and Author Disclosures

This study was supported in part by a Grant-in-Aid for Scientific Research from the Ministry of Education, Science and Culture of Japan (18590815 and 21590898) and a Grant in Aid of Japanese Arteriosclerosis Prevention Fund. Dr Higashi has reported that he has no relationships relevant to the contents of this paper to disclose.
